# Can single molecule localization microscopy be used to map closely spaced RGD nanodomains?

**DOI:** 10.1371/journal.pone.0180871

**Published:** 2017-07-19

**Authors:** Mahdie Mollazade, Thibault Tabarin, Philip R. Nicovich, Alexander Soeriyadi, Daniel J. Nieves, J. Justin Gooding, Katharina Gaus

**Affiliations:** 1 EMBL Australia Node in Single Molecule Science, School of Medical Sciences and the ARC Centre of Excellence in Advanced Molecular Imaging, University of New South Wales, Sydney, Australia; 2 School of Chemistry, Australian Centre for NanoMedicine and the ARC Centre of Excellence in Convergent Bio-Nano Science and Technology, University of New South Wales, Sydney, Australia; LAAS-CNRS, FRANCE

## Abstract

Cells sense and respond to nanoscale variations in the distribution of ligands to adhesion receptors. This makes single molecule localization microscopy (SMLM) an attractive tool to map the distribution of ligands on nanopatterned surfaces. We explore the use of SMLM spatial cluster analysis to detect nanodomains of the cell adhesion-stimulating tripeptide arginine-glycine-aspartic acid (RGD). These domains were formed by the phase separation of block copolymers with controllable spacing on the scale of tens of nanometers. We first determined the topology of the block copolymer with atomic force microscopy (AFM) and then imaged the localization of individual RGD peptides with direct stochastic optical reconstruction microscopy (dSTORM). To compare the data, we analyzed the dSTORM data with DBSCAN (density-based spatial clustering application with noise). The ligand distribution and polymer topology are not necessary identical since peptides may attach to the polymer outside the nanodomains and/or coupling and detection of peptides within the nanodomains is incomplete. We therefore performed simulations to explore the extent to which nanodomains could be mapped with dSTORM. We found that successful detection of nanodomains by dSTORM was influenced by the inter-domain spacing and the localization precision of individual fluorophores, and less by non-specific absorption of ligands to the substratum. For example, under our imaging conditions, DBSCAN identification of nanodomains spaced further than 50 nm apart was largely independent of background localisations, while nanodomains spaced closer than 50 nm required a localization precision of ~11 nm to correctly estimate the modal nearest neighbor distance (NDD) between nanodomains. We therefore conclude that SMLM is a promising technique to directly map the distribution and nanoscale organization of ligands and would benefit from an improved localization precision.

## Introduction

The nanoscale organization of the extracellular environment influences cellular behaviors such as adhesion, migration and differentiation [1][2]. In particular, cell adhesion and spreading are highly sensitive to the nanoscale spatial organization of adhesive ligands such as the tripeptide arginine-glycine-aspartic acid (RGD) found in some extracellular matrix (ECM) proteins [3][4]. Using patterned surfaces with well-defined chemistries, previous work identified a critical spacing of adhesive ligands in the range of 30–80 nm that controlled effective adhesion, spreading of cells and formation of focal adhesions [5][6][7][8]. However, the actual distribution of adhesive ligands was often not directly measured and had to be inferred from the design of the surface modifications. For example, electron microscopy or atomic force microscopy (AFM) [9][10][11] can determine the underlying topology of engineered surfaces. However, it has been challenging to directly map the nanoscale distribution of ligands with these techniques so that the ligand presentation can be linked to the organization of cell surface receptors in cells that adhere to these surfaces [12][13] [14].

In principle, single molecule localization microscopy (SMLM) techniques, such as (fluorescent) photoactivated localization microscopy ((f)PALM) [15][16] and (direct) stochastic optical reconstruction microscopy ((d)STORM) [17][18], point accumulation for imaging nanoscale topography (PAINT [19][20][21][22]), and ground-state depletion followed by individual molecule return (GSDIM)[23] could address this challenge. In SMLM, the position of single molecules is recorded by stochastically switching fluorophores between a stable dark state and a short-lived fluorescent state. With organic fluorophores, localisation precisions of 10–20 nm can easily be achieved [24]. To date, SMLM has been mainly used as an imaging tool in biology [25][26][27][28] while the use of SMLM to analyze and quantify surface patterns and chemistries is still at its infancy [29][30][31]. This is because SMLM yields discreet molecular coordinates of individual molecules rather than images and thus requires further analysis to parameterize, for example, ligand distribution into nanodomains. Here, we explore the suitability of a cluster analysis called DBSCAN (density-based spatial clustering application with noise) [32] to detect the RGD nanodomains on nanopatterned surfaces from SMLM data. The experimental data was supplemented with simulations to identify the imaging and surface parameters that influenced the ability and reliability of nanodomain detection.

## Results and discussion

**Fig 1:**
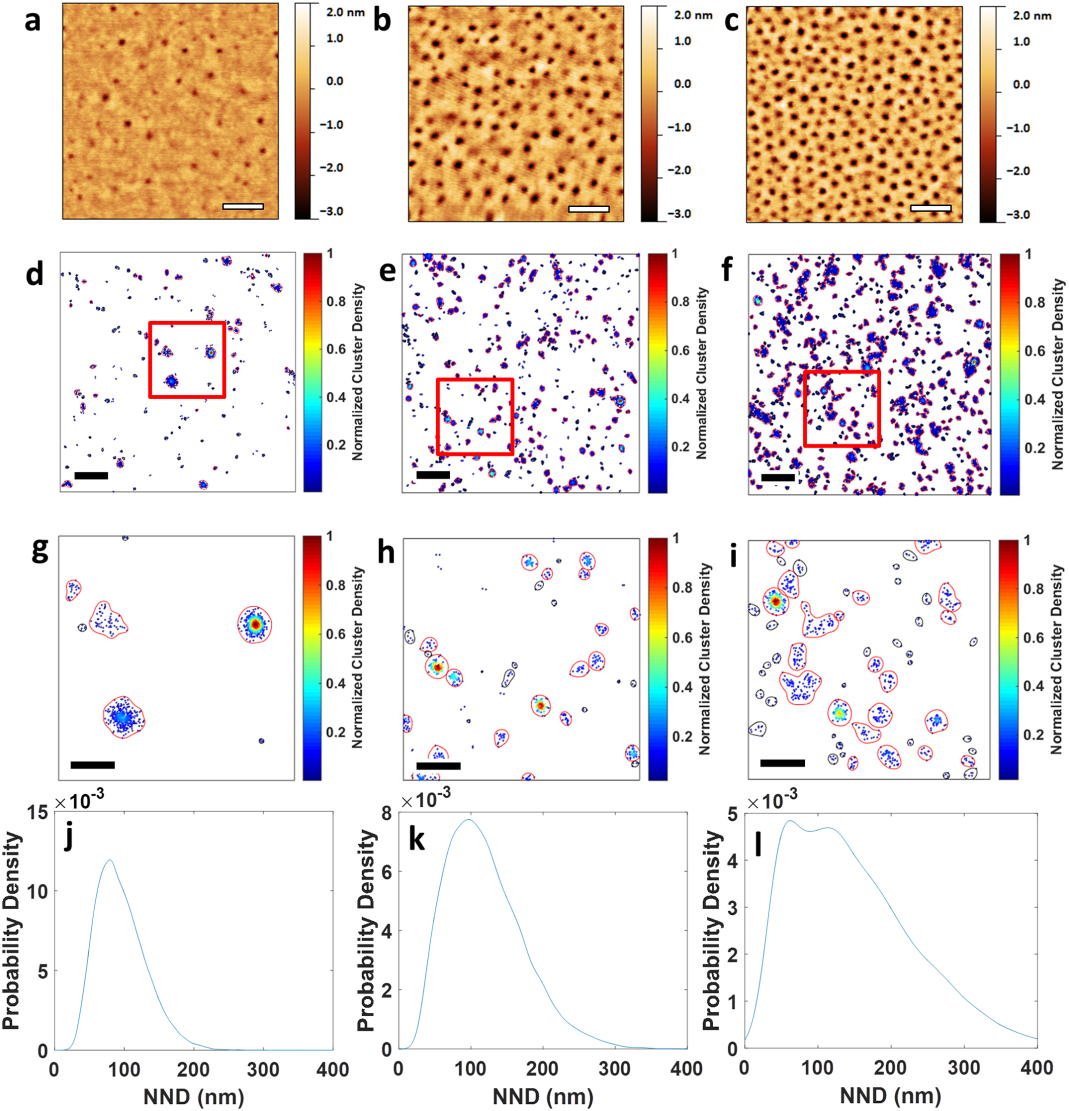
AFM and dSTORM images of nanoscale surface with different PS:PS-PEO ratios. **a-c. AFM images for surfaces containing 25:75 PS-PEO:PS**. (A), 50:50 PS-PEO:PS (B) and 100:0 PS-PEO:PS (C), respectively. Scale bars = 100 nm. (D-J). Cluster density maps from dSTORM data of CGRGDSK-Alexa647 on surfaces containing 25:75 PS-PEO:PS (D and G), 50:50 PS-PEO:PS (E and H) and 100:0 PS-PEO:PS (F and I), respectively. dSTORM images in (G-I) are zoomed regions of the highlighted red squares in (D-F), respectively. Color-coding represented molecular density. Scale bars in D-F = 500 nm; scale bars in G-I = 100 nm. Contours enclosing clusters are red if the cluster possesses the minimum number of points for the DBSCAN analysis, whereas those in black do not. For AFM imaging 3 images were taken of 2 independent repeats of each surface preparation. For each different surface preparation 3 images were taken and used for dSTORM analysis. J-L. Distribution of nearest neighbor distances (NND) between cluster centers identified by DBSCAN (with ε = 20 nm and minPts = 3) of CGRGDSK-Alexa647 dSTORM images on 25:75 PS-PEO:PS (J), 50:50 PS-PEO:PS (K) and 100:0 PS-PEO:PS (L) surfaces, respectively.

The phase separation of block copolymers [33] can be exploited to create nanodomains with varying diameters and interdomain spacings that are on the biologically relevant scale of tens of nanometers [34][35][36][37][38]. For example, a polystyrene-poly(ethylene oxide) (PS-PEO) copolymer system [39] was used to engineer a controlled cellular microenvironment for mesenchymal stem cells that contained nanodomains decorated with cell adhesion peptides [40]. We have produced surfaces where vertical cylinders of PEO in thin films of PS-PEO:PS mixtures on glass coverslips formed nanodomains [41] that were easily detectable as ~ 3 nm indentations with AFM (Fig 1A–1C, Figure A (B-C) in S1 File). Changing the PS-PEO to PS ratio resulted in different nearest neighbor distances (NND) between the domain centers, i.e., 58 (± 14) nm, 38 (± 3) nm and 31 (± 1) nm for 25:75 PS-PEO:PS, 50:50 PS-PEO:PS and 100:0 PS-PEO:PS surfaces, respectively, while the domain diameters were maintained at ~10 nm (Table 1). The prior functionalization of the PEO group with a maleimide group enabled the attachment of cysteine-terminated RGD containing peptides, CGRGDSK, conjugated to the fluorophore AlexaFluor647 (RGD-AF647) to the surface. This enables dSTORM imaging and the localization of RGD-AF647 molecules with an average localization precision of 16 nm (Figure B (D) in S1 File).

**Table 1:** Average cluster area, density and NND from AFM and dSTORM imaging of surfaces with different PS-PEO:PS-PEO ratios.

	Average NND (nm)		Average diameter (nm)
PS:PS-PEO	AFM	dSTORM	AFM
**25:75**	58 ± 14	57 ± 9	10 ± 1
**50:50**	38 ± 3	30 ± 1	10 ± 1
**100:0**	31 ± 1	29 ± 3	10 ± 1

We applied a cluster method DBSCAN [32] to the dSTORM data that detects clusters of points using a propagative strategy based on two parameters; ε, the search radius and minPts, the minimum number of neighbours within this search radius (Figure C in S1 File). As expected, RGD-AF647 molecules were located in nanoscale clusters (Fig 1). However, dSTORM clusters were fewer in number and larger in size than the PEO cylinders determined by AFM. A direct comparison between AFM and dSTORM data may not be valid: AFM is a direct measure of the nanotopology of the surface, and thus all domains, while dSTORM requires the domains to be labelled with a fluorophore. Therefore, if the coupling efficiency of RGD-AF647 molecules to PEO cylinders is low, this would result in fewer nanoclusters detected in dSTORM than the nanodomains detected in AFM [31]. Similarly, the differences in average domain sizes measured with AFM and dSTORM may be explained with the localization uncertainty inherent to dSTORM. Fluorophores located at the edge of a domain could only be localized within the 16 nm localization precision, meaning that dSTORM clusters could appear approximately 18 nm larger (FWHM, $\sigma \sqrt{2\text{ln}\left(2\right)}$) in radius than the AFM nanodomains. This raises the central question of this study: can analysis of dSTORM data map the RGD spatial distribution without having to infer domain geometry from the underlying polymer topology? A complicating factor for this endeavor is the presence of background fluorescence that could be caused by fluorescent species other than RGD-AF647 molecules that were present in the polymer surface and buffers (Figure D (A-B) in S1 File), or non-specific absorption of RGD-AF647 molecules onto the polymer surfaces (Figure D (C-D) in S1 File).

As a first assessment of the suitability of nanodomain detection, we calculated the nearest-neighbor distance (NDD) for DBSCAN-identified RGD-AF647 clusters. Since the clusters were round, we opted for measuring NDD from cluster center to cluster center, which is appropriate for the circular clusters observed here. This measure does not take cluster size into account and for non-circular domains NDD determination from cluster edge to cluster edge may be more appropriate. The modal NND obtained by AFM (58 ± 14 nm) and dSTORM (57 ± 9 nm) were in agreement for the 25:75 PS:PS-PEO surface, indicating that NDD values were a useful measurement. The 50:50 PS:PS-PEO and 100:0 PS:PS-PEO surfaces had modal NNDs of 96 ± 1 nm, and 78 ± 8 nm respectively with DBSCAN and 38 ± 3 nm and 32 ± 1 nm respectively with AFM (Table 1,Fig 1J–1L), illustrating that closely spaced domains may be challenging to map with dSTORM.

**Fig 2:**
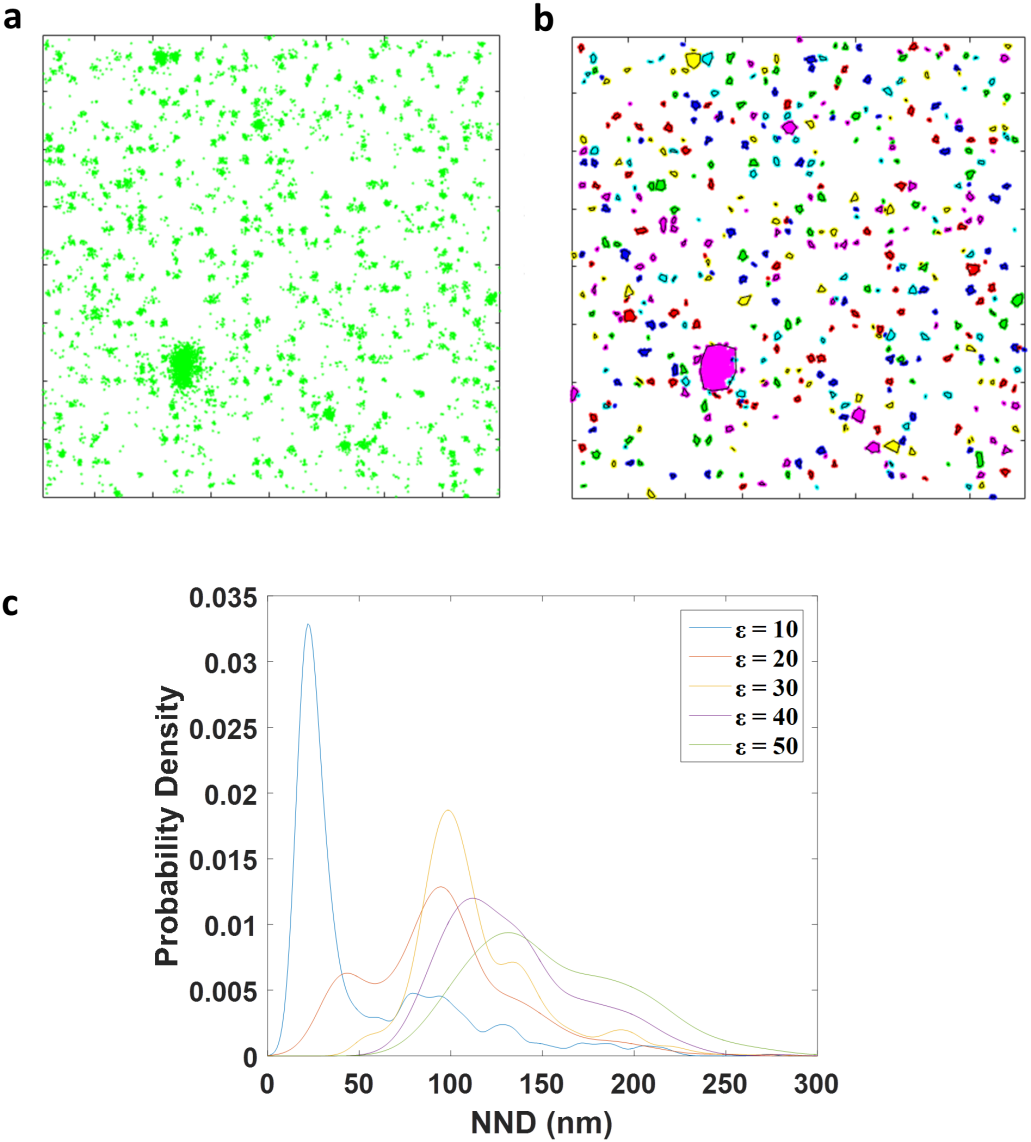
DBSCAN and NND analysis of simulated nanodomain surface. (A) Simulated dSTORM image with nanodomain of 10 nm in diameter and spaced 100 nm apart. Background was added to the simulated nanodomains by randomly choosing image regions of the experimental data of non-specific absorption data (Figure D (B-D) in S1 File) and with 75% of missing domains. (B) Individual clusters, represented by different colors and black contour lines, identified by DBSCAN (parameters; ε = 20 nm and minPts = 3) of the simulated data set shown in (A). (C). Distribution curves of nearest neighbor distances (NDD) of simulated data shown in (A-B) for various DBSCAN search parameters, ε.

**Fig 3:**
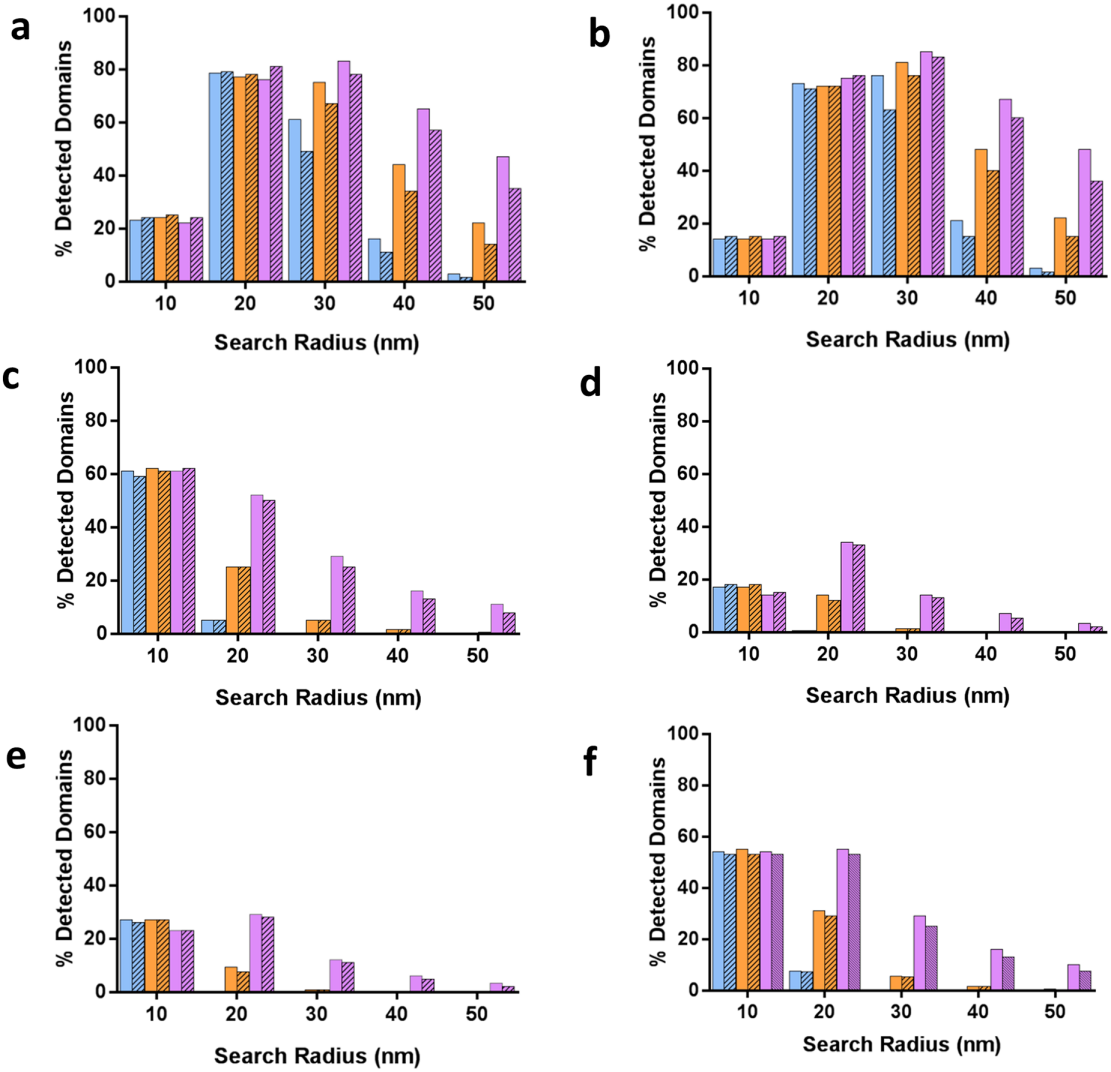
Effect of the domain spacing, presence of background, percentage of missing nanodomains and localization precision on nanodomain detection in simulated data by DBSCAN. Each simulated nanodomain was constructed around a central point, which corresponds to its true center. After identification of clusters by DBSCAN, the contour of each identified cluster was tested against the true center of the simulated domain. A cluster was counted as correctly detected if its contour contained one and only one true center. (A-F). Percentage of correctly detected clusters for different search radii (ε = 10 to 50 nm) and values of minPts = 3 (A, C, E) and minPts = 5 (B, D F) with 0% (blue), 50% (orange) and 75% (purple) of missing domains with (striped) or without (solid) included background. In a-b, domain spacing was set to 100 nm. In (C-F), domain spacing was set to 50 nm. In (A-D), the log-normal distribution of the localization precision centered at 16 nm (μ = 2.8 and σ = 0.28; matching the experimental data, Figure B (D) in S1 File); in (E-F), the log-normal distribution of the localization precision centered at 11 nm (μ = 2.4 and σ = 0.28, Figure B (D) in S1 File).

**Fig 4:**
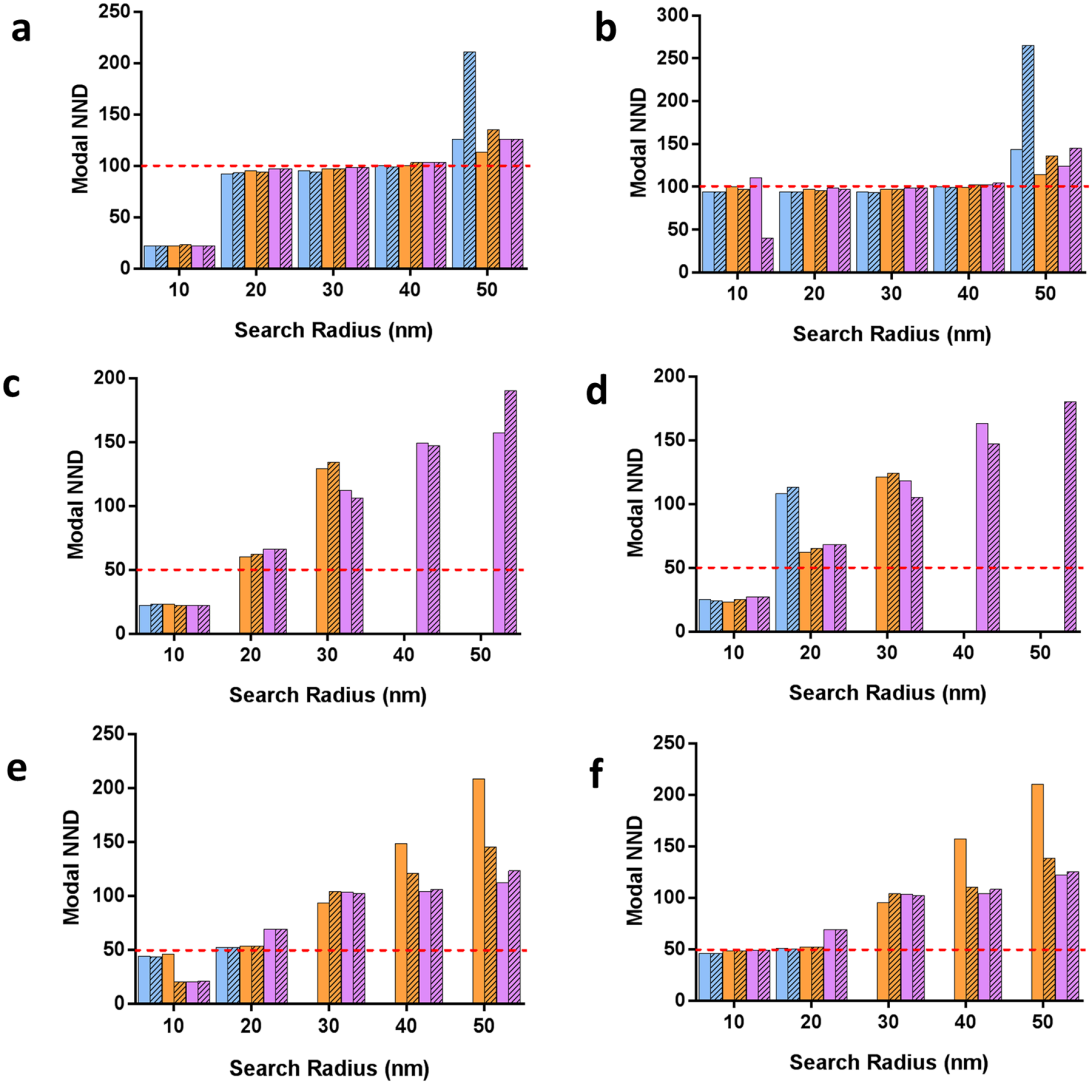
Effect of the domain spacing, presence of background, percentage of missing nanodomains and localization precision on NND between detected nanodomains by DBSCAN in simulated data. Simulation conditions were identical to Fig 3. From the NND distributions, the highest occurrence for the NND was extracted and plotted. (A-F) Modal NNDs for different search radii (ε = 10–50 nm) and minPts values of 3 (A, C, E) and 5 (B, D F), with 0% (blue), 50% (orange) and 75% (purple) of undetected domains and with (striped) or without (solid) included background. In (A-B), domain spacing was set to 100 nm. In (C-F), domain spacing was set to 50 nm. In (A-D), the log-normal distribution of the localization precision centered at 16 nm (μ = 2.8 and σ = 0.28; matching the experimental data, Figure B (D) in S1 File); in (E-F), the log-normal distribution of the localization precision centered at 11 nm (μ = 2.4 and σ = 0.28; Figure B (D) in S1 File

To understand how dSTORM data influences NND values, we simulated dSTORM data that mimicked surfaces with nanodomains that had interdomain distances of 100 nm and 50 nm (Fig 2A) and systematically varied the DBSCAN parameters ε and minPts in the subsequent analysis (Figs 3 and 4). The simulated images were generated from a surface model in which the number of molecules per domain was defined by a Poissonian distribution (μ = 15 and σ = 4) centered on an estimated value of 15 molecules per domain [42] (Figure B (A) in S1 File). Also included in the model were the experimentally derived, intrinsic blinking properties of AlexaFluor647 under dSTORM imaging conditions [43], which were well described by a geometric distribution (*η*^*n*^(1−*η*)), n is the number of blinking events, with the best fit for the parameter η = 0.5 [44] (Figure B (B) in S1 File). Localization precision for each blinking event was determined by sampling an empirical distribution of localization precisions from a bare glass surface sparsely covered with AlexaFluor647 molecules [45][46]. We estimated the precision of localization for each blinking event as a log-normal distribution with mean μ = 2.8 and standard deviation σ = 0.28 (Figure B (C) in S1 File). We introduced a parameter corresponding to the percentage of missing domains that could be used to adjust the level of incomplete coupling of fluorophores to PEO cylinders or undetected fluorophores [31]. Instead of simulating background, we drew from dSTORM images of polymer surfaces that did not present maleimide moieties, but were incubated with the RGD-AF647. Any signal from these surfaces should be from non-specifically absorbed fluorophores and erroneous background detection events. For each simulation, an area of the size of the simulation was randomly chosen from a pool of measured background images and included with the background-free simulated nanodomains. It should be noted that we selected these dSTORM imaging parameters for our simulations as they most closely matched the experimental observations. However, other microscopes, fluorophores and experimental conditions may result in other photophysical properties and thus substantially different dSTORM data sets. Thus, the values derived from the simulations, such as optimal DBSCAN parameters and NDD distributions, are specific to these data sets while the methods and general conclusions are more widely applicable.

The simulated detection event coordinates were analyzed by DBSCAN with different search radii, ε, and the NDD distributions plotted (Fig 2). As was the case with experimental data, the NND distribution of simulated images had a clear maximum. This modal NND was at the expected value of 100 nm for ε = 20 nm and ε = 30 nm when domains were spaced 100 nm apart (Fig 2). However, this was not the case for ε = 40 and 50 nm, most likely because nanodomains were not correctly identified when the search radius was either too small or too large.

To test this hypothesis, we varied the DBSCAN search radius (ε = 10 to 50 nm) and the minPts parameter (minPts = 3 and minPts = 5), used two values for domain spacing (50 nm and 100 nm), varied the percentage of missing domains (0%—blue, 50%—orange, 75%—purple), and compared data with and without background (solid *versus* striped). In each case we quantified the efficiency of nanodomain detection by calculating the percentage of domains that are correctly detected (Fig 3). Surprisingly, we found that the DBSCAN search radius of ε = 20 nm made domain detection insensitive to the presence of background, missing fluorophores and the minPts parameter when domains were spaced 100 nm apart, with domain detection efficiency remaining at 76–81% (Fig 3A and 3B). In contrast, background and incomplete detection impacted severely on correct identification of clusters when larger search radii were used (Fig 3A and 3B) or domains were spaced 50 nm apart *versus* 100 nm (Fig 3C and 3D). A closer inspection of the simulated images revealed that the smallest search radius of ε = 10 nm fragmented the simulated domains into additional clusters, accounting for the low cluster detection efficiency seen in Fig 3A–3D (Figure E in S1 File). This fragmentation is a consequence of the stochastic and sparse point sampling of the underlying sample, characteristic of a SMLM imaging experiment and the DBSCAN algorithm, which segments regions of locally high density. It is then necessary to ensure that a DBSCAN search radius is sufficiently large to avoid these fragmentation artefacts. However, excessively large search radii (e.g., ε = 50 nm) could lead to the merging of domains into large clusters (Figure E in S1 File). Increasing the DBSCAN minPts from 3 to 5 had relatively little effect on cluster identification but improved the correct identification for ε = 30 nm for 100 nm interdomain spacing, increasing detection efficiency to 63–85% (Fig 3B) because fewer domains were merged into large clusters. Overall, we identified that a high density of closely spaced domains as the main limiting factor for domain detection with this approach, but domains with 100 nm spacing can be robustly detected with a DBSCAN radius of ε = 20 nm.

We used the simulation to examine how cluster detection could be experimentally improved when domains are spaced only 50 nm apart. We reduced the simulated mean localization precision from 16 nm (Fig 3C and 3D) to 11 nm (Fig 3E and 3F, Figure B (D) in S1 File), resulting from a change in the log-normal distribution parameter μ = 2.8 to μ = 2.4. As a result, we observed a remarkable improvement in the detection efficiency for clusters spaced 50 nm apart at ε = 10 nm and ε = 20 nm, with approximately 60% and 54% of domains detected, respectively (Fig 3E and 3F). At ε = 10 nm the results were again insensitive to the level of background, the density of domains and the minPts parameter. At ε = 20 nm, however, as the percentage of missing domains decreased, the number of detected domains decreased as well (Fig 3E). If a localization precision of 11 nm could be achieved experimentally, it would be possible to correctly identify nanodomains with 50 nm spacing, as seen with more than 50% of domains detected by using the DBSCAN parameters of ε = 10–20 nm.

For simulated domains spaced 100 nm apart (Fig 4A and 4B), the NND distributions peaked at the expected value for ε = 20 to 40 nm and thus provides a more reliable measure than direct identification of individual domains. The NND peak position was again insensitive to the density of domains, presence of background and the minPts parameter. A search radius of ε = 10 nm resulted in a grossly underestimated modal NND value (22 nm) while ε = 50 nm yielded a slightly overestimated modal NND value (113–135 nm), except when high background and a high density of domains were present.

When domains were spaced 50 nm apart, where less than 40% of domains were correctly identified, the modal NND was extremely dependent on the search radius (Fig 4C and 4D). Only for ε = 20 nm did the NND value of 60–66 nm come close to the expected value of 50 nm when 50% or 75% domains were undetected. An improvement in the localization precision rescued the modal NND position at 46–49 nm and 50–52 nm for ε = 10 nm and ε = 20 nm, respectively, for domains that were spaced 50 nm apart, particularly when minPts = 5 (Fig 4E and 4F) and when fewer than 75% domains were undetected.

**Fig 5:**
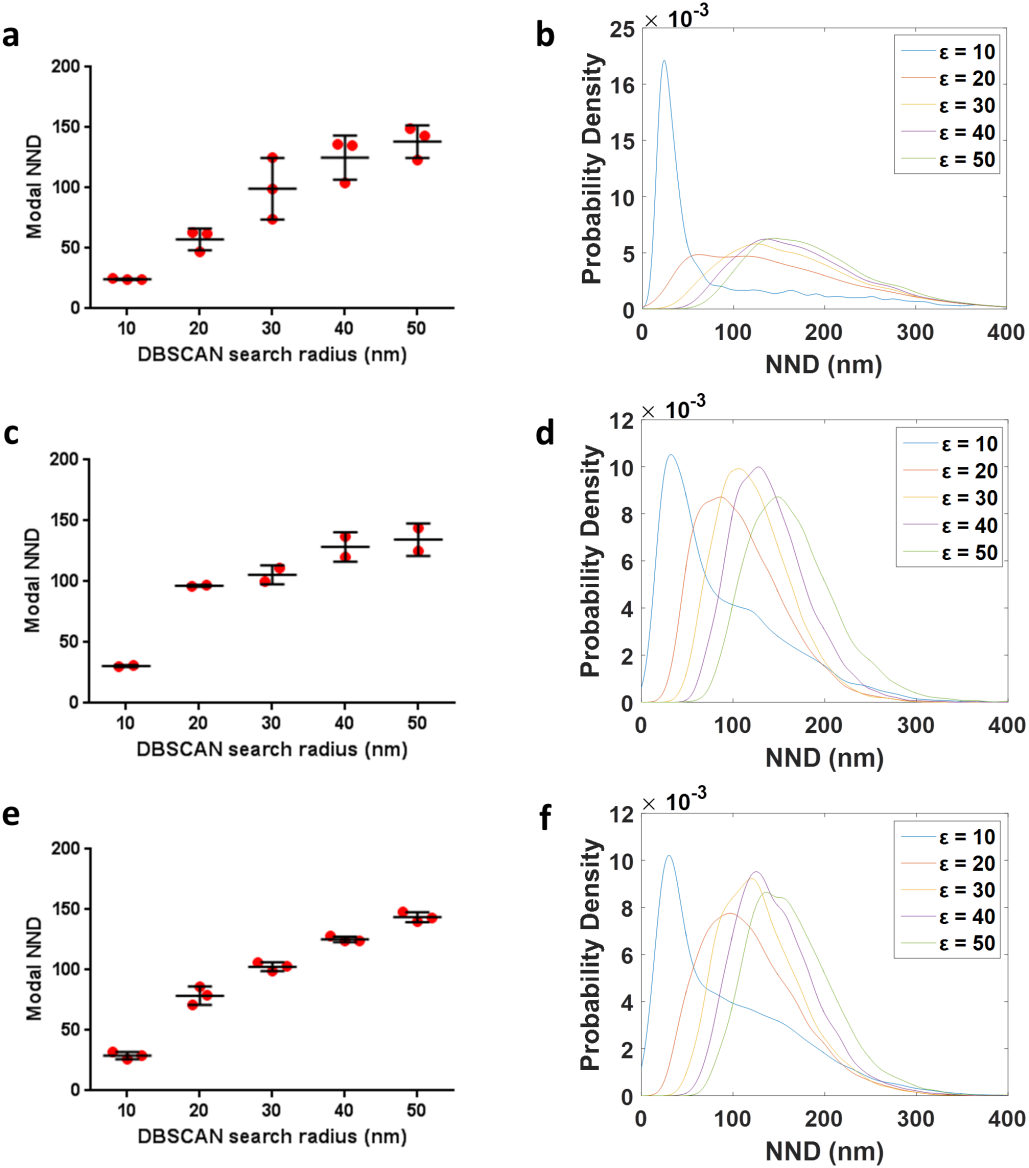
NND analysis of RGD-AF647 clusters on phase separated polymer surfaces. (A-F). Modal NND (A, C, E) and representative NND distributions (B, D, F) for different DBSCAN search radii (r = 10–50 nm) for RGD-AF647-modified surfaces containing 25:75 PS-PEO:PS (A-B), 50:50 PS-PEO:PS (C-D) and 100:0 PS-PEO:PS (E-F), respectively. In (A), (C) and (E), data are mean and standard deviation of 3 independent measurements.

With the insights from the simulations, we re-examined the NDD distributions for three polymer surfaces of 25:75 PS-PEO:PS (Fig 5A and 5B), 50:50 PS-PEO:PS (Fig 5C and 5D) and 100:0 PS-PEO:PS (Fig 5E and 5F). The DBSCAN search radius influenced the NND distribution and modal NND for all surfaces. This was expected given that the simulations revealed that cluster detection and NND results were strongly influenced by high domain density and short interdomain spacing. The simulation results indicated that the best estimate for the 25:75 PS-PEO:PS surface would be provided by ε = 20 nm, which yielded a modal NND of 57 ± 9 nm, closely in agreement with the interdomain distance measured by AFM of 58 ± 14 nm. Given the excellent agreement between the dSTORM NND and AFM NND, we concluded that non-specific adsorption and incomplete labeling and detection of domains with RGD-AF647 molecules was not the major obstacle.

The interpretation of the NND dSTORM analysis for 50:50 PS-PEO:PS surfaces (Fig 5C and 5D) and 100:0 PS-PEO:PS surface (Fig 5E and 5F) was not so straightforward. The AFM interdomain distances were 37 ± 3 nm and 31 ± 1.3 nm, respectively. Ana lysis of corresponding dSTORM data with the NND approach using a search radius value of ε = 10 nm yielded modal NND values of 30 ± 1 nm and 29 ± 3 nm, respectively. It is thus possible that under these conditions, the small DBSCAN search radius correctly distinguished the interdomain distance. However, for ε = 20 nm, we observed NND values above 75 nm for 50:50 PS-PEO:PS surfaces (Fig 5C and 5D) and 100:0 PS-PEO:PS surfaces (Fig 5E and 5F). Drawing the comparison to the simulated results, the very high density of points overall caused the misidentification of clusters by DBSCAN. As the simulation suggested an improvement in localization precision may result in more reliable NND values at high density, indicating that it would be more beneficial to improve the localization precision than the domain labeling protocols.

## Conclusion

The nanoscale distribution of adhesive ligands is key to the formation and maturation of focal adhesions. Therefore, materials whereby the distribution of adhesive ligands, such as the RGD peptide, can be controlled at the nanometer scale are important tools for probing focal adhesion formation. However, directly mapping ligand distribution has remained challenging. We were motivated to explore whether dSTORM and an appropriate analysis could be used for mapping even closely spaced RGD nanodomains, thus providing a route to RGD ligand mapping in the presence of cells seeded on the engineered surface.

A number of dSTORM cluster analyses have been reported in the literature in addition to the DBSCAN method mentioned here, including methods based on mesh representations [47][48][49] or on Ripley’s functions [50]. An implementation of the latter approach includes an extension by which appropriate segmentation parameters are chosen via a Bayesian engine. Similar to the work here, a parameter space is sampled and resulting clusters evaluated against an expected model for cluster morphology to determine an optimal segmentation. The Bayesian approach requires a model for the spatial distribution of clusters; in the demonstrated work this is Gaussian clusters amongst spatially random background points, which may be well-suited for the system here. The DBSCAN approach, however, does not require an *a priori* model for cluster shape and, in contrast to any Ripley’s function-based methods, can deal with clusters of non-circular shape.

The optical properties of the PS-PEO:PS block copolymer enabled dSTORM imaging of RGD-AF647 and the identification of nanoscaled adhesive domains that were spaced ~58 nm apart. Importantly, the analysis still robustly determined the average interdomain spacing despite factors such as background signal, incomplete domain labeling and missing domains. Determination of interdomain spacing became difficult when the simulated domains were spaced 50 nm or less apart. Encouragingly, by increasing the apparent localization precision of the labeling by 30% (from mean localization precision of 16 nm to 11 nm) it becomes possible to regain domain identification. Therefore, we conclude that dSTORM in combination with DBSCAN analysis is a promising route for characterization of the nanoscale features of block copolymer domain surfaces with closely spaced domains. As pointed out above, the values for interdomain spacing that could be reliably detected with dSTORM and DBSCAN were specific to our imaging and simulation parameter, while the general conclusion should be applicable to other engineered surfaces and imaging conditions: the main limiting factor in mapping closely spaced nanodomains with dSTORM and DBSCAN is the localization accuracy and detection efficiency of the domain. Improved dSTORM imaging buffers, such as cyclooctatetraene containing buffer [51], could mean a greater percentage of domain labels could be detected and therefore improve identification. Moreover the use of techniques that can increase the number of photons collected, such an interference dual objective system [52][53] or adaptive optics [54][55], and minimizing drift during acquisition, e.g., stabilized TIRF [56][57], would give additional improvements and push the limit of domain identification. This would in turn allow a direct far-field approach to observing and characterizing of ligand distributions on nanodomain-patterned surfaces.

## Materials and methods

### Materials

Polystyrene-block-polyethylene oxide (PS-PEO) copolymer (with a molecular weight of 190 kDa for the PS block and a molecular weight of 48 kDa for the PEO block) and polystyrene (PS) polymers of 11.4 kDa were purchased from Polymer Source. HPLC grade toluene was purchased from Ajax Finechem. Microscope glass cover slides (high precision with low background, No 1.5 H) were purchased from Australian Scientific. Thiolated-RGD peptides conjugated with AlexaFluor647 (Ac-CGRGDSK (AF647)-amide) were supplied by Cambridge Research Biochemicals. Tris (2-carboxyethyl) phosphine) (TCEP) and *N*-(p-maleimidophenyl) isocyanate (PMPI) were purchased from Thermo Scientific. HEPES and EDTA were supplied by Life Technologies, Australia. Dry *N*,*N*-dimethylformamide (DMF), PBS and all other materials were sourced from Sigma-Aldrich and used as supplied.

### Hydrophobic substrate preparation and spin casting

Microscope glass cover slides were rinsed thoroughly in MilliQ water and then exposed to UV/ozone (UV Ozone Cleaner, Model UV.TC.220, Bioforce Nanosciences) for 20 min to remove any organic molecules. In a post ozone treatment, the cover slides were rendered hydrophobic by incubation in boiling benzyl alcohol (BnOH) for 4 h, followed by thorough rinse in isopropanol and dried under a stream of nitrogen gas. Thin films of polymer were produced by spin casting of blends of polymer solutions containing 1% (wt/vol) total polymer concentration in toluene onto the BnOH-treated cover slides at different rpm values.

### Atomic force microscopy

The surface topography of the thin layer of polymer on the cover slides was characterized by atomic force microscopy (AFM) using a MultiMode 8-HR AFM (Bruker, Santa Barbara, CA) in air at room temperature. AFM was operated in tapping mode using Scanasyst-Air cantilevers (Bruker) with a nominal spring constant of 0.4 N.m^-1^ and a resonance frequency of 70 kHz. For each sample, AFM images of at least 5 individual areas (500 nm × 500 nm) were acquired. The collected images were analyzed by a custom-written MATLAB script to determine the interdomain distance and the size of the domains. A built-in MATLAB function “im2bw” was used to convert the AFM images into binary maps with a threshold at 20% above the average intensity of the image. Thereafter binary images were segmented with the built-in MATLAB function “bwonncomp” to identify the connected pixels that form domains. Finally, the built-in MATLAB function “regionprops” was used to determine the centre and the area of each domain.

### Maleimide functionalization of PS-PEO polymer

100 mg of PS-PEO copolymer and 8 mg of PMPI were dissolved in 2 mL of anhydrous DMF by heating up to 40°C for 1 hour. After removing DMF through rotary-evaporation, the solid product was re-dissolved in chloroform and then precipitated into cold methanol. The precipitate was recovered through multiple cycles of vacuum filtration and washing with methanol to remove any un-reacted PMPI.

### ^1^H NMR of derivatised polymer

^1^H High-resolution NMR spectra were recorded using a Bruker Avance III 600 MHz spectrometer or Bruker Avance III 300 MHz employing CDCl_3_ as the solvent. Sample concentration was approximately 20 mg mL^-1^. Chemical shifts were referenced to the residual proton peak of CDCl_3_ at 7.26 ppm. 64 scans were accumulated per increment over a spectral width up to 8.5 ppm. ^1^H NMR in CDCl_3_ for PS-PEO modified with maleimide demonstrated two additional peaks (Bruker Avance III 600 MHz, δ = 7.76 d 2H, δ = 7.53 d 2H) compared to ^1^H NMR in CDCl3 for PS-PEO (Bruker Avance III 300 MHz NMR), which was an indicator of the successful addition of maleimide.

### RGD immobilization to maleimide-functionalized polymer

Thiolated RGD peptides (Ac-CGRGDSK (AF647)-amide) conjugated with AlexaFluor647 were bound to the maleimide groups of the polymer layer spin cast onto cover slides. This was achieved by incubating 5 μg mL^-1^ RGD peptide in coupling buffer (0.1 M TCEP, 0.2 M HEPES, 1 mM EDTA, pH 7.2) with the cover slides for 1 hour at room temperature. The surfaces were then washed thoroughly in PBS.

### dSTORM imaging

The dSTORM principle and instrumentation have been previously described [18][17]. Our dSTORM imaging system is a total internal reflection fluorescence microscope (ELYRA; Zeiss) with a 100x oil-immersion objective (NA = 1.46). For activation and excitation of AlexaFluor647, we used lasers at 405 nm and 642 nm, respectively. During the acquisition, the 642 nm laser was used at 15 mW for imaging and the 405 nm laser set in the range 10–100 μW was used for the conversion of AlexaFluor647 molecules from the dark state. The fluorescence emission was obtained by acquiring 20,000 images per sample on an electron-multiplying charged-coupled device camera (iXon DU-897; Andor) using an exposure time of 30 ms. To perform dSTORM, the samples were imaged in an oxygen-scavenging PBS-based buffer containing 25 mM HEPES, 25 mM glucose, 5% glycerol, 0.05 mg mL^-1^ glucose oxidase and 0.025 mg mL^-1^ horseradish peroxidase, supplemented with 50 mM cysteamine; all from Sigma. The buffer in combination with high laser intensity increased the dark state lifetime of AlexaFluor647, promoting stochastic blinks of fluorescent molecules required for dSTORM imaging. At the end of an acquisition, the raw data consisted of a stack of 20,000 raw fluorescence intensity images that were processed subsequently.

### dSTORM data processing

Raw fluorescence intensity images were processed using Zen 2011 SP3 Software (Zeiss MicroImaging). After Gaussian filtering, in each acquired frame, true events were separated from the camera residual background when I-M > 6S [58], where I is the event intensity, M the mean image intensity and S the standard deviation of the image intensity. Each true event corresponding to a point-spread function is fitted to a two-dimensional Gaussian distribution to calculate its center, accounting for the possibility of overlapping peaks. The localization precision was calculated based on the definition of Mortensen *et al* [45]. To correct for potential sample drift, we used a built-in drift correction function from the Zen software that uses a method based on the triangulation of localizations and a piecewise-linear drift model. Finally, tables containing the *x-y* particle coordinates of each spot detected in the acquisition were generated and used subsequently for DBSCAN analysis.

### DBSCAN and NND analysis

For quantification of dSTORM data, we used density-based spatial clustering application with noise analysis (DBSCAN) [32] and nearest neighbor distance (NND). DBSCAN identifies clusters in large datasets of points by a propagative method that links points belonging to a common cluster based on two parameters; r, the search radius and ε, the minimum number of neighbors. These two criteria define whether a point belongs to a cluster or not; if a point has at least ε neighbors within a radius ‘r’ or if a point is a neighbour of a point belonging to a cluster, this point is assigned to the cluster. The unassigned points are assigned to noise. The DBSCAN routine was implemented in MATLAB but the core algorithm was coded in C++ and compiled in a MEX file to improve the speed of processing. NND analysis uses the center of each cluster identified by DBSCAN and, using a built-in MATLAB function using a k d tree algorithm, calculates the distance between the center of a cluster and the center of the nearest cluster.

### Simulations

We performed the simulations using a custom-written MATLAB script. Each simulated region was 5 μm x 5 μm. First, we created an array of points corresponding to the centers of the domains separated by 100 nm or 50 nm. Then, each domain was independently constructed around each point. For each domain, we randomly assigned a number of virtual molecules contained in this domain using a Gaussian distribution with mean, μ = 15 and standard deviation, σ = 4. Molecules were randomly and uniformly positioned in a 5 nm radius around the center, thereby simulating a 10 nm domain diameter. Each molecule was then assigned a number of blinks from a geometric distribution and a localization precision from a log-normal distribution. It should be noted that the average time in the fluorescent on-state is relative to the camera’s integration time and here we assumed a geometric distribution. The localization precision defines the sigma of the 2D Gaussian function for the distribution of blinks around the molecule of interest. In a recent report, Lee et al. determined that the blinking properties of a fluorescent probe in SMLM conditions were best described by a geometric distribution with the parameter η = 0.5, corresponding to the probability of the transition to the dark state [44]. The log-normal distribution of localization precisions was calculated from the fit of AlexaFluro647-coated coverslip data in dSTORM conditions. For each blinking event, we recorded the photon count, which can be converted into precision localization values [46][45]. The best fit for AlexaFluor647 molecules was obtained for μ = 2.8, σ = 0.28. We added background to the simulations by superimposing a randomly selected area of 5 μm x 5 μm from the non-specific binding adsorption data (Figure D (C) in S1 File). Finally, to simulate improperly labeled domains or undetected domains, we introduced a percentage of missing domains where we randomly removed a specific percentage of the domains.

## Supporting information

S1 File Figure A: AFM and dSTORM images of nanoscale surfaces produced by copolymer self-assembly. Figure B: Defining the dSTORM parameters employed in simulation: number of molecule per domain, number of blink per molecule, precision per event. Figure C: DBSCAN and NND analysis for dSTORM images of nanoscale surfaces. Figure D: dSTORM image of background and non-specific binding of CGRGDSK-Alexa647 onto surfaces modified with 25:75 PS-PEO:PS polymer. Figure E: DBSCAN cluster maps of simulated data.
(PDF)
