# Supplementary material for: Can single molecule localization microscopy be used to map closely spaced RGD nanodomains?
Source: PLoS One. 2017 Jul 19;12(7):e0180871. doi: 10.1371/journal.pone.0180871 (PMC5516992; doi:10.1371/journal.pone.0180871)
Supplement: S1 File — Figure A: AFM and dSTORM images of nanoscale surfaces produced by copolymer self-assembly. Figure B: Defining the dSTORM parameters employed in simulation: number of molecule per domain, number of blink per molecule, precision per event. Figure C: DBSCAN and NND analysis for dSTORM images of nanoscale surfaces. Figure D: dSTORM image of background and non-specific binding of CGRGDSK-Alexa647 onto surfaces modified with 25:75 PS-PEO:PS polymer. Figure E: DBSCAN cluster maps of simulated data. (PDF) [file pone.0180871.s001.pdf]

Mapping nanoscale domains of ligands to cell surface receptors by single-molecule localization microscopy

Mahdie Mollazade[1¶], Thibault Tabarin[1¶], Philip R. Nicovich[1,#a], Alexander Soeriyadi[2], Daniel J. Nieves[1], J. Justin Gooding[2], and Katharina Gaus[1 *]

[1]EMBL Australia Node in Single Molecule Science, School of Medical Sciences and the ARC Centre of Excellence in Advanced Molecular Imaging, University of New South Wales, Sydney, Australia.

[2]School of Chemistry, Australian Centre for NanoMedicine and the ARC Centre of Excellence in Convergent Bio-Nano Science and Technology, University of New South Wales, Sydney, Australia.

[#a] Current Address: Allen Institute for Brain Science, Seattle, Washington, United States of America.

[¶] These authors contributed equally to this work

*Corresponding Author

E-mail: k.gaus@unsw.edu.au (KG)

SUPPORTING INFORMATION

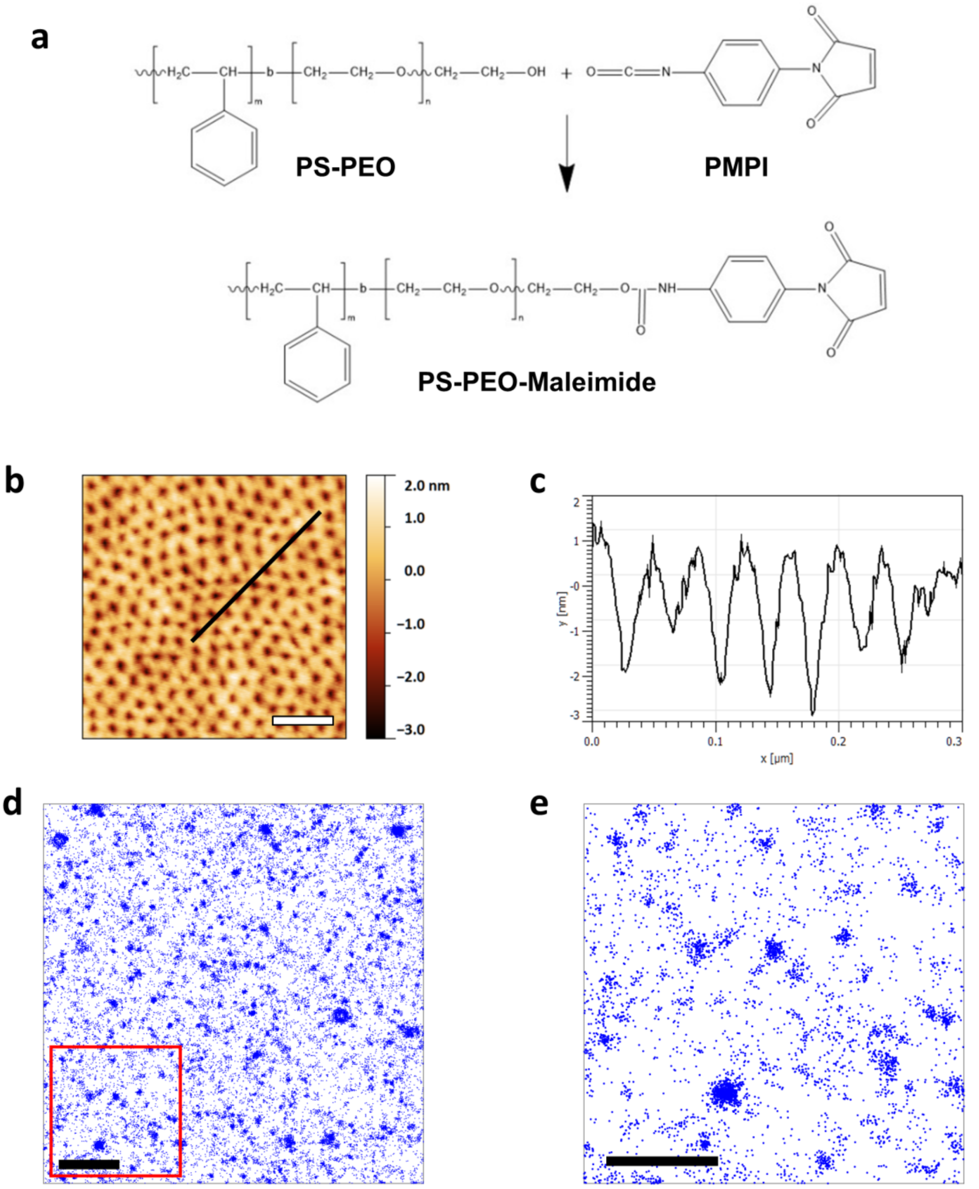

Figure A. AFM and dSTORM images of nanoscale surfaces produced by copolymer self-assembly.

(A) The copolymer system consisted of a mixture polystyrene (PS) and poly(styrene-ethylene oxide) (PS-PEO). PS-PEO block was conjugated to maleimide by a substitution nucleophilic type 2 (SN2) reaction between the alcohol group from PEO and the cyanate from N-(p-Maleimidophenyl)isocyanate (PMPI). (B) Representative AFM image of the polymer mixture of 100:0 PS-PEO:PS spin-coated onto a glass surface. AFM images were acquired in tapping mode. Scale bar = 100 nm. (C) Profile of the polymer surface along the line profile taken in b (black solid line). (D-E) Representative dSTORM image of the CGRGD-Alexa647 molecules coupled to a 100:0 PS-PEO:PS phase-separated polymer surface via the maleimide linker. Each localization is represented with a dot. (E) is a zoomed region of the highlighted square (red) in d. Scale bar in d = 1 µm; scale bar in e = 500 nm. The experiment for each condition was repeated at least 3 times.

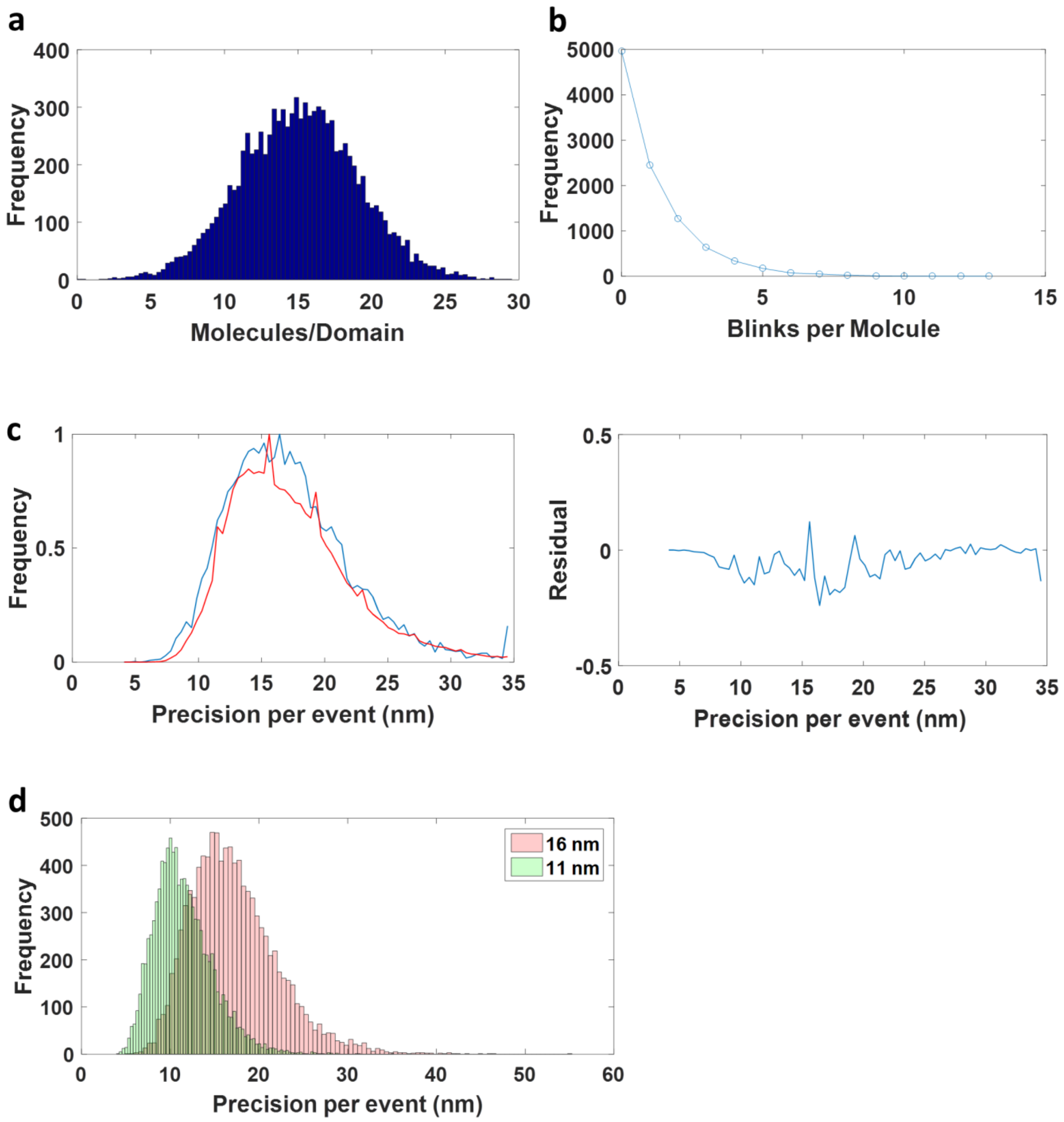

Figure B. Defining the dSTORM parameters employed in simulation: number of molecule per domain, number of blink per molecule, precision per event.

(A) Distribution of the number of molecules per domain using a Poissonian distribution with mean and standard deviation, $\mu = 15$ and $\sigma = 4$, respectively. (B) Distribution of number of blinks per molecule with a geometric distribution with the parameter $\eta = 0.5$ corresponding to the probability of the transition to the dark state. (C) Distribution of localization precision per blink. Red curve represents experimental data of AlexaFluor 647 molecules absorbed onto a coverslip. Blue curve represents the best fit using log-normal distribution with logarithm of the mean and the standard deviation, $\mu = 2.8$ and $\sigma = 0.28$ with the residuals of the fit (right panel). (D) Comparison of the distributions of two localization precisions used in the simulation. Parameters extracted from the best fit best of data shown in (C) with the center of the distribution at 16 nm (red). A distribution was generated with an improved localization precision, $\mu = 2.4$, $\sigma = 0.28$, centered at 11 nm (green).

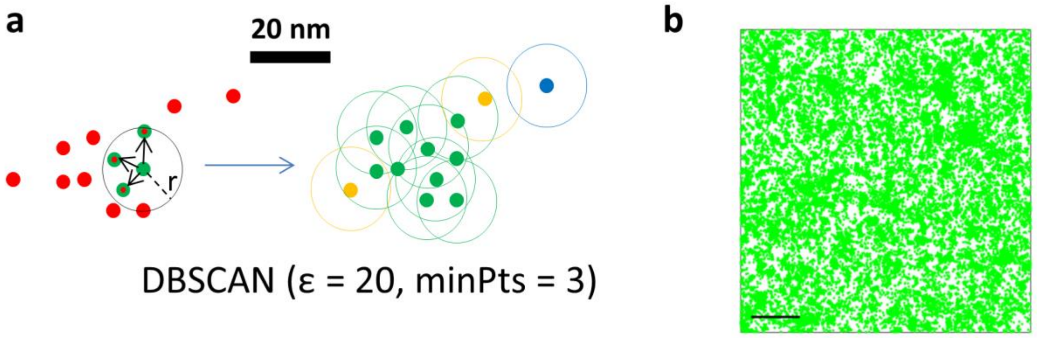

Figure C. DBSCAN and NND analysis for dSTORM images of nanoscale surfaces.
(A) Schematic of DBSCAN. DBSCAN is a propagative cluster detection method where points are connected if the number of neighbor is equal or above a certain threshold (e.g. minPts = 3) within a radius (e.g., ε = 20 nm). If the conditions are fulfilled the points are labelled as belonging to a cluster (green dots) and the connection propagates until the conditions are broken (yellow dots). Any points not associated to a cluster are labelled as noise (blue dots). (B) dSTORM image of CGRGDSK-Alexa647 conjugated to a 25:75 PS-PEO:PS surface. Scale bar = 1 μm.

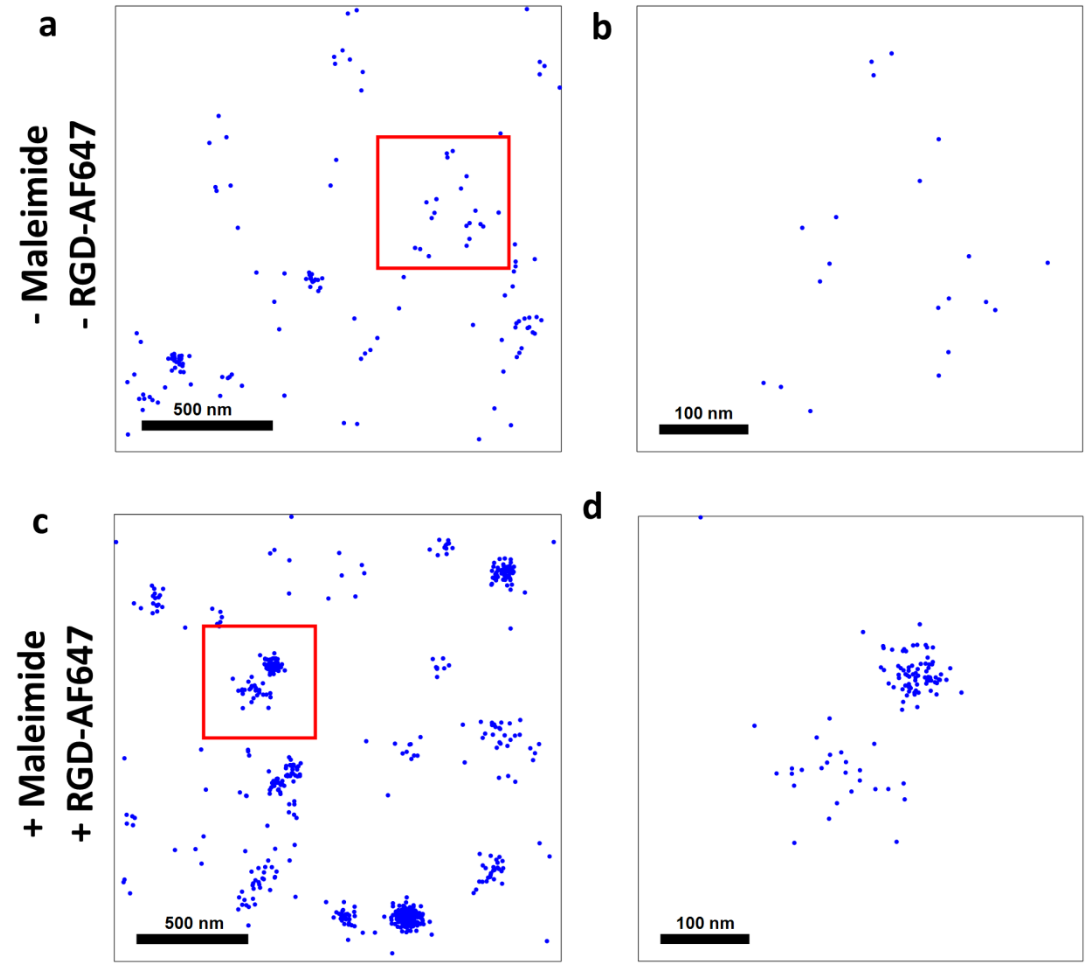

Figure D. dSTORM image of background and non-specific binding of CGRGDSK-Alexa647 onto surfaces modified with 25:75 PS-PEO:PS polymer.

(A-B) dSTORM image of background fluorescence on nanodomain surfaces without maleimide modification and without exposure to CGRDGSK-Alexa647 molecules. (C-D) dSTORM image of non-specific absorption onto nanodomain surface without maleimide modification but with exposure to CGRDGSK-Alexa647 molecules. b and d are zoomed regions defined by the red square in a and b respectively. Scale bars in (A) and (C) = 500 nm; scale bars in (B) and (D) = 100 nm. The average densities of events were 232 localizations/µm2 (without CGRGDSK-AlexaFluor647) and 395 localizations/μm^2 (with CGRGDSK-AlexaFluor647), suggesting a high level of background fluorescence. Molecules other than CGRGDSK-AlexaFluor647 only resulted in little randomly distributed localizations (S4A-B Fig).

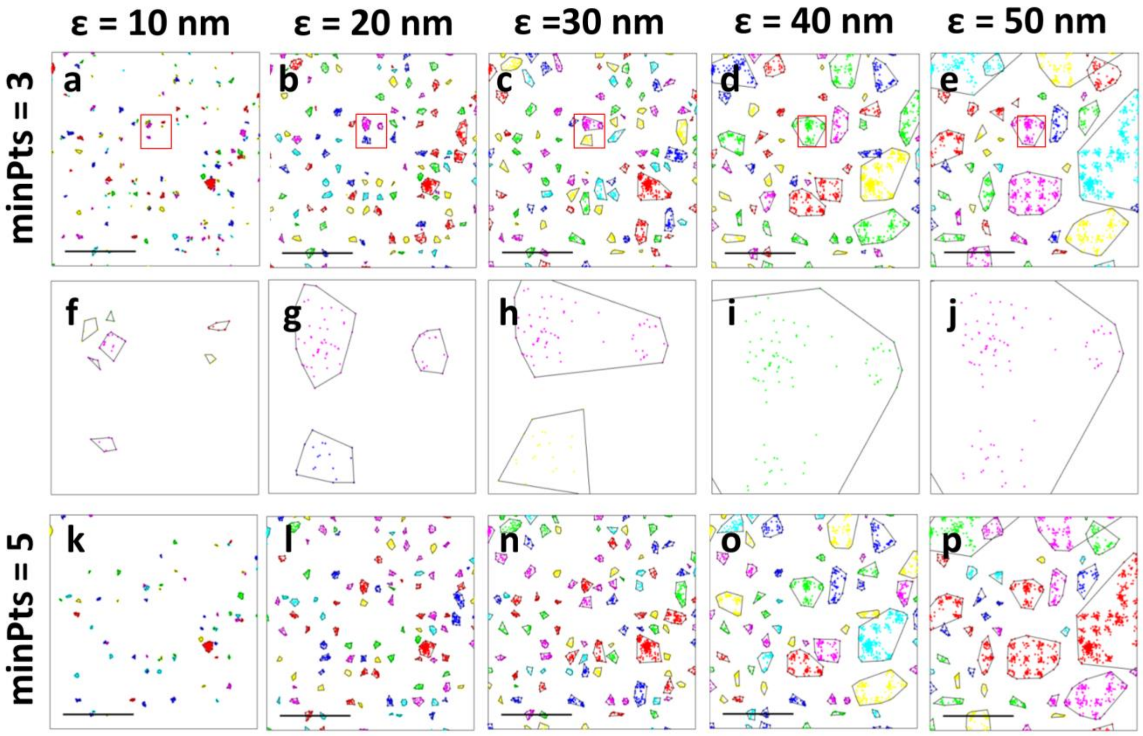

Figure E. DBSCAN cluster maps of simulated data.

Cluster maps for simulated data shown in Fig 5 and Fig 6, with 100 nm interdomain distances, with background and 75 % domains undetected. Each cluster identified by DBSCAN was color-coded and given a black contour. DBSCAN search parameter, ε, was varied from 10 to 50 nm, with the minPts parameter set at 3 (**A-E**) or 5 (**K-P**). (**F-J)** Zoomed regions correspond to highlighted regions (red square) from (**A-E)** respectively.
